# Trends in malignant neoplasm of bone and articular cartilage related mortality among older adults in United States (1999–2020)

**DOI:** 10.1097/MS9.0000000000002629

**Published:** 2024-09-30

**Authors:** Abdul Haseeb, Rana Muhammad Umer Nasrullah, Mohammad Arham Siddiq, Hafsah Alim Ur Rahman, Syed Muhammad Sinaan Ali, Damni Advani, Laksh Kumar, Muhammad Ashir Shafique, Muhammad Saqlain Mustafa, Sandesh Raja, Adarsh Raja, Khabab Abbasher Hussien Mohamed Ahmed

**Affiliations:** a Jinnah Sindh Medical University, Karachi, Pakistan; b Dow University of Health Sciences, Karachi, Pakistan; c Liaquat National Hospital and Medical College, Karachi, Pakistan; d Shaheed Mohtarma Benazir Bhutto Medical College Lyari, Karachi, Pakistan; e Faculty of Medicine, University of Khartoum, Khartoum, Sudan

**Keywords:** articular cartilage, bone, CDC WONDER, epidemiology, malignant neoplasm

## Abstract

**Introduction:**

Malignant neoplasms of bone and articular cartilage, although rare, are associated with substantial morbidity and mortality, posing a serious health burden. Understanding the trends in mortality related to these cancers is crucial for developing targeted interventions and improving patient outcomes. This study aims to analyze long-term mortality trends, identify demographic and geographic disparities, and uncover potential factors driving changes in mortality rates.

**Methods:**

This retrospective study analyzed mortality rates among individuals aged 65 and older from 1999 to 2020 using CDC WONDER death certificate data, abstracting demographics, geographic factors, and urban/rural status.

**Results:**

From 1999 to 2020, 18,205 adults aged 65 and older died from malignant neoplasms of bone and cartilage. The age-adjusted mortality rate (AAMR) started at 20 per 100,000 in 1999 and steadily declined until 2012 (APC: −1.12). However, from 2012 onwards, there was a notable reversal, with the AAMR rising sharply to 23.8 by 2020 (APC: 4.73). Men had higher mortality rates than women, with NH Black individuals showing the highest rates among races. Southern states and nonmetropolitan areas had elevated AAMRs, suggesting targeted interventions for better outcomes and lower death rates.

**Conclusion:**

The findings highlight significant inequities, with Southern states and nonmetropolitan areas showing elevated age-adjusted mortality rates (AAMRs). These geographic disparities underscore the urgent need for targeted public health interventions in these regions to improve cancer outcomes and reduce mortality. Addressing these gaps is essential for achieving more equitable health outcomes, particularly in high-risk populations.

## Introduction

Highlights

- Malignant neoplasms of bone and articular cartilage are rare but impactful cancers with significant morbidity and mortality rates.
- Understanding mortality trends and identifying influencing factors are crucial for improving patient outcomes and public health interventions.
- Eighteen thousand two hundred five fatalities from malignant neoplasms of bone and articular cartilage in adults aged 65 and older from 1999 to 2020.
- Highest age-adjusted mortality rate (AAMR) was 23.8 in 2020.
- Men had higher AAMRs (25.7) compared to women (15.6), and NH Black or African American individuals had the highest AAMRs (20.6) among racial groups.
- Study provides insights into mortality trends of bone and cartilage malignant neoplasms in older adults.

Malignant tumors of bone and articular cartilage constitute a relatively rare category of neoplasms, accounting for less than 0.2% of all malignancies^1^. Bone tumors can greatly affect patients’ morbidity and mortality due to their potential to invade locally, destroy bone tissue, and metastasize to distant locations. Risk factors for malignant bone tumors include ionizing radiation, genetic disorders like Li-Fraumeni and Rothmund-Thompson syndromes, and hereditary retinoblastoma. Other factors include specific bone diseases like Paget’s and osteomyelitis^2,3^. Advancements in diagnosis and treatment have led to improved outcomes for osteosarcoma patients, although age, sex, and height remain significant factors^4^. Surgical resection is the primary treatment option, with chemotherapy and neoadjuvant therapies used in some cases^4^. Recent molecular and genetic research has enabled targeted therapies, resulting in increased success rates for previously untreatable osteosarcomas, with 5-year overall survival rates approaching 70–80%^5,6^. Despite advancements in diagnosis and treatment, significant disparities remain in outcomes based on demographic factors such as age, sex, and ethnicity^7,8^.

Studying demographic and regional trends in adults older than 65 with bone and articular cartilage cancer is crucial due to the unique challenges this population faces. Osteosarcoma, though more common in younger individuals, also affects older adults, with about 1 in 10 cases occurring in those over 60. In patients aged 75 and above, diagnosis is complicated by nonspecific radiological and histological findings and delayed consultations, making early detection difficult^9^. Individualized treatment, including surgery and radiotherapy, is essential for maintaining the quality of life, especially since these tumors often develop in critical areas like the arms, legs, or pelvis^10^. Understanding these trends helps identify at-risk groups and guide targeted interventions, improving outcomes for this vulnerable population.

Given the limited research on the mortality trends of primary malignant bone and articular cartilage tumors, this study aims to fill this gap by analyzing population-based registry data from 1999 to 2020. The study will investigate mortality rates and explore the impact of demographic and geographic factors, providing critical insights for public health strategies and resource allocation.

## Methods

### Study setting and population

In order to determine the mortality rates for malignant neoplasms of bone and articular cartilage in people 65 years of age and older between 1999 and 2020, the study used death certificate information from the CDC WONDER database. The CDC WONDER database provides a comprehensive collection of mortality data, drawn from death certificates across all 50 states and the District of Columbia, ensuring a robust national representation. Data were accessed through the CDC WONDER online system, which allows users to query a wide range of public health data based on various criteria, including cause of death, geographic location, and demographic factors. The system provides data in a deidentified format, which preserves privacy while enabling detailed epidemiological analysis. Malignant neoplasms of bone and articular cartilage were identified in the study using ICD-10 codes C40 and C41, which are provided for administrative databases^6^. These codes specifically refer to malignancies of the bone and articular cartilage, and cases were included if these codes appeared as either the primary or secondary cause of death on the certificate. The data were further filtered to include only individuals aged 65 and older, to focus on the older adult population, which is at higher risk for these types of malignancies. Mortality rates were calculated using age-adjusted rates per 100 000 persons to account for differences in age distribution across the population over time. These calculations were performed using standard population data from the U.S. Census Bureau, which is integrated within the CDC WONDER system, allowing for accurate trend analysis across the study period. The research did not require any approval from the institutional review board since it followed STROBE reporting requirements and used deidentified public use data from the government.

### Data abstraction

Data was extracted on multiple variables, including demographic information (age, sex, and race/ethnicity), geographic factors (state and region), site of death, and urban/rural classification. People passed away in a variety of places, including homes, hospices, hospitals, and long-term care institutions. Statistics on sex, age, race, and ethnicity were collectively referred to as ‘demographics’. The categories for race and ethnicity were as follows: non-Hispanic Whites, non-Hispanic Blacks or African Americans, non-Hispanic Latinos, non-Hispanic American Indians or Alaska Natives, non-Hispanic Asians, and non-Hispanic Pacific Islanders. The information came from death certificates, which were previously used in studies with the WONDER database^11^. Urban areas were classified into two groups under the National Centre for Health Statistics Urban-Rural Classification Scheme: big metropolitan areas, which have a population of one million or more, and medium/small metropolitan areas, which have a population between 50 000 and 999 999.

Less than 50 000 people lived in rural areas, with more counties being established by the U.S. Census conducted in 2013^12^. According to US Census Bureau guidelines, the Northeast, Midwest, South, and West were among the geographic divisions^13^.

### Statistical analysis

We conducted a detailed statistical analysis of mortality rates for malignant neoplasms of bone and articular cartilage, both in unadjusted and age-adjusted forms, from 1999 to 2020. The 95% CIs for each of the following rate categories were given: year, sex, race/ethnicity, state, and urban/rural status. Crude mortality rates were calculated by dividing the total number of deaths by the corresponding US population for each year. Age-adjusted mortality rates (AAMRs) were standardized to the 2000 US population to account for age distribution differences over time^14^.

To analyze changes in mortality at the national level, the annual percent change (APC) and 95% CI in AAMR were calculated using the Joinpoint Regression Program (Version 5.0.2, National Cancer Institute)^15,16^. Joinpoint regression is particularly suited for this type of analysis because it can detect multiple changes in trend direction within the data, rather than assuming a constant rate of increase or decrease. This is crucial for accurately identifying periods of significant change, such as abrupt increases or decreases in mortality rates, that may correspond to factors like advancements in medical treatment or changes in disease prevalence^17^. This statistical method uses log-linear regression models to identify points where a significant change in trend occurs, allowing us to pinpoint shifts in mortality rates over time. Two-tailed *t*-test was used to establish the significance of the slope, which allowed for the classification of the changes in mortality as either increasing or decreasing. *P*-values below 0.05 were considered statistically significant.

## Result

**Figure 1:**
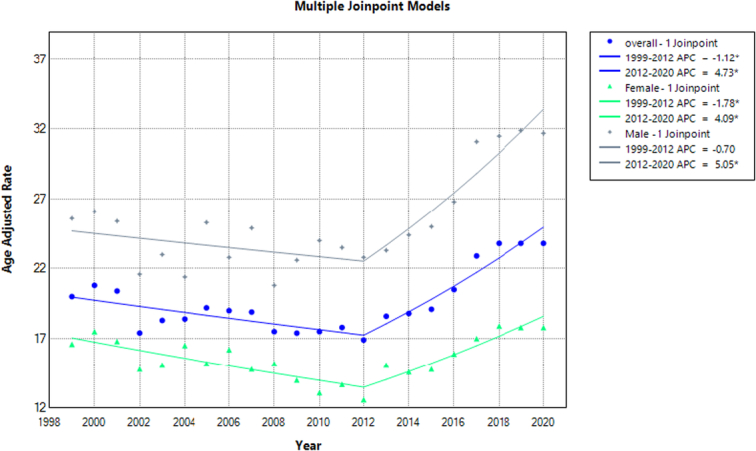
Mortality rates for malignant neoplasms of bone and articular cartilage, stratified by. Sex in adults aged 65 years or older in the United States between 1999 and 2020, along with the associated age-adjusted mortality rates (AAMRs) per 1 000 000. The * denotes the annual percentage change (APC) that was found to be statistically significant at α=0.05.

Between 1999 and 2020, malignant neoplasm of bone and articular cartilage caused 18 205 deaths in 92 847 666 persons 65 years of age or older. Eight thousand five hundred fifty-two females and 9653 males were among them (Fig. 1, Supplemental Table 1, http://links.lww.com/MS9/A609). Out of 16 351 deaths for whom the place of death was documented, 18.37% happened in hospitals, 29.9% in long-term care or nursing homes, 7.7% at hospices, and 45.1% at home (Supplementary Table 2, http://links.lww.com/MS9/A609).

### Annual trends in mortality for malignant neoplasm of bone and articular cartilage

Overall, The AAMR for malignant neoplasm of bone and articular cartilage-related deaths in adults was 20 in 1999, which rose to 23.8 at the end of 2020. There was a decrease in the number of deaths from 1999 to 2012, with (APC: −1.12; 95% CI: −2.44 to −0.22). This was followed by an increase in trends from 2012 to 2020, with (APC: 4.73; 95% CI: 2.99–8.49). The highest AAMR of 23.8 (95% CI: 22.5–25.2) was recorded in 2020 (Fig. 1, Supplementary Tables 3,4, http://links.lww.com/MS9/A609).

### Malignant neoplasm of bone and articular cartilage related mortality, AAMR stratified by sex

**Figure 2:**
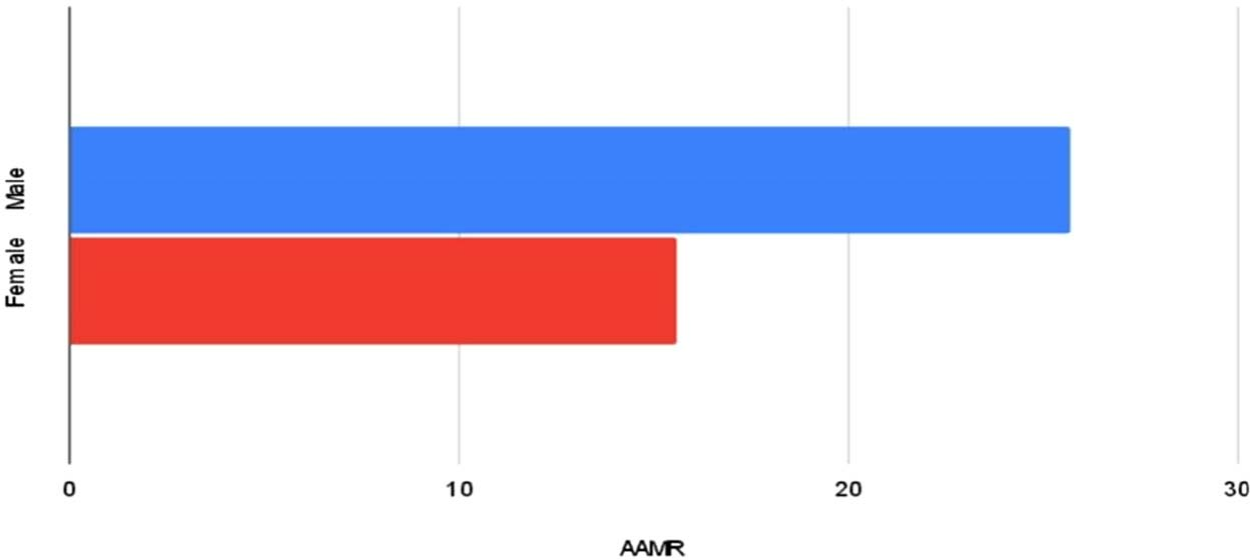
Bar graph demonstrating overall AAMRs value among males and females from 1999 to 2020 (overall AMMR men=25.7, women=15.6).

Men have higher AAMRs than women during analyzing years, with an overall average of 25.7 for men (95% CI: 25.2–26.2) and 15.6 for women (95% CI: 15.3–15.9), see Figure 2. Starting from 1999, men’s AAMRs were 25.6 (95% CI: 22.9–28.4), which gradually declined until 2012 to 22.8 (APC: −0.70, 95% CI: −3.51 to 0.46). Thereafter, there was a steep rise in AMMRs until 2020, with the highest slope in 2019 at 31.9 (APC 5.05; 95% CI: 2.61–13.30). For women, there was a gradual decrease in AMMRs until 2012, reaching 12.6 (APC: −1.78; 95% CI: −3.50 to −0.82). After this period, there was a steep rise in trends until 2020, with the highest AMMRs of 17.9 (APC: 4.09; 95% CI: 2.04–9.43) in 2018 (Fig. 1, Supplementary Table 3,4, http://links.lww.com/MS9/A609).

### Malignant neoplasm of bone and articular cartilage related mortality, AAMR stratified by race

**Figure 3:**
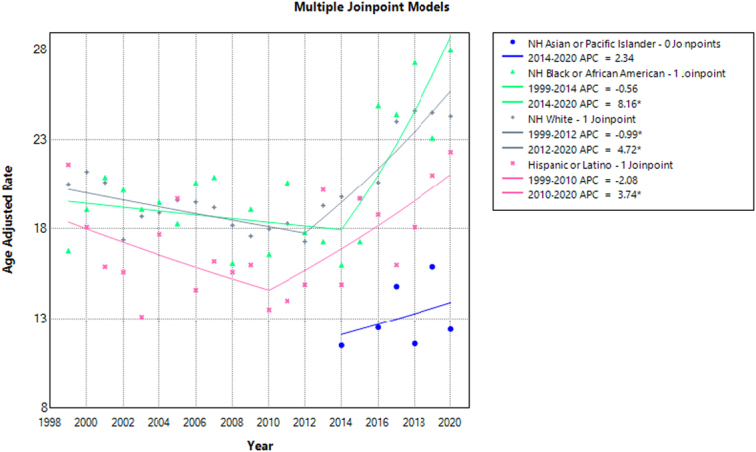
Mortality rates for malignant neoplasms of bone and articular cartilage, stratified by. Race in adults aged 65 years or older in the United States between 1999 and 2020, along with the associated age-adjusted mortality rates (AAMRs) per 1 000 000. The * denotes the annual percentage change (APC) that was found to be statistically significant at α=0.05.

The NH Black or African American, NH White, NH American Indian or Alaska Native, NH Hispanic or Latino, and NH Asian or Pacific Islanders had the greatest values in the AAMR stratification based on race. The overall AAMR for NH Black or African American was 20.6 (95% CI: 19.6–21.6), for NH White it was 20.2 (95% CI: 19.9–20.5), for NH American Indian or Alaska Native, it was 19.1 (95% CI: 15.1–23.8), for Hispanic or Latino it was 17.5 (95% CI: 16.5–18.5), and for NH Asian or Pacific Islanders it was 10.5 (95% CI: 9.30–11.6). A decrease in trend was observed in NH White from 1999 to 2012 (APC: −1.02; 95% CI: −2.15 to −0.22). Afterwards, there was a steep rise in death rates from 2012 to 2020 (APC: 4.70; 95% CI: 3.24–7.15) among NH whites. Similar trends were observed in NH Black or African American with a decrease in trends from 1999 to 2014 (APC: −0.48; 95% CI: −9.79 to 1.57) and the highest death rates in 2020 (APC: 7.80; 95% CI: 2.09–25.05). Similar trends were also observed in Hispanic or Latino from 1999 to 2012 (APC: −2.13; 95% CI: −15.48 to 0.91) and 2012–2020 (APC: 3.72; 95% CI: 1.36–14.60). NH Asian or Pacific Islander had increased death rates from 2014 to 2020 (APC: 2.28; 95% CI: −7.99 to 16.58). However, the APC for NH American Indian or Alaska Native was unknown due to the unavailability of data (Fig. 3, Supplementary Tables 3,5, http://links.lww.com/MS9/A609).

### Malignant neoplasm of bone and articular cartilage related mortality, AAMR stratified by geographical location:

**Figure 4:**
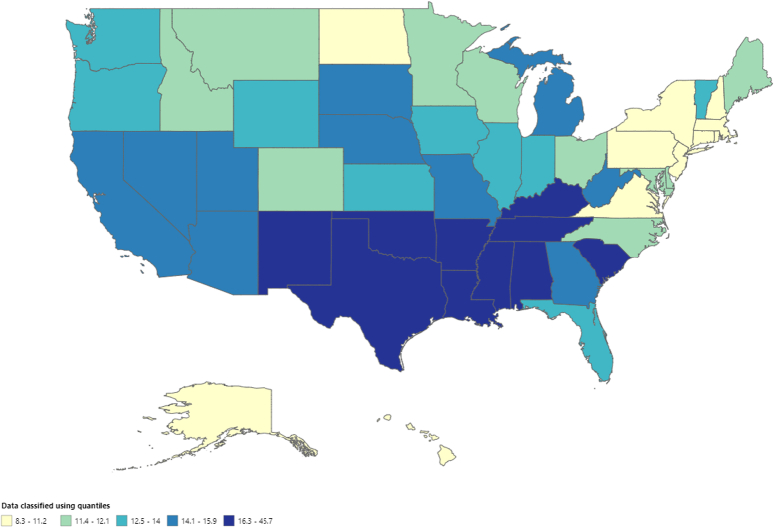
Mortality rates of malignant neoplasm of bone and articular cartilage, stratified by states in adults (age 65 years or greater) in the United States from 1999 to 2020, along with age-adjusted mortality rates per 1 000 000 among states (ranging from 8.3 to 45.7).

The AAMRs of the states varied widely, ranging from 12.0 (95% CI: 9.0–15.7) in Hawaii to 65.0 (95% CI: 59.5–70.4) in Mississippi. In states with death rates in the 90th percentile, such as Alabama, Arkansas, and Mississippi, AAMRs were five times higher than in states with low 10th percentile death rates, such as the District of Columbia, Connecticut, North Dakota, and New Hampshire (Fig. 4, Supplementary Table 6, http://links.lww.com/MS9/A609).

**Figure 5:**
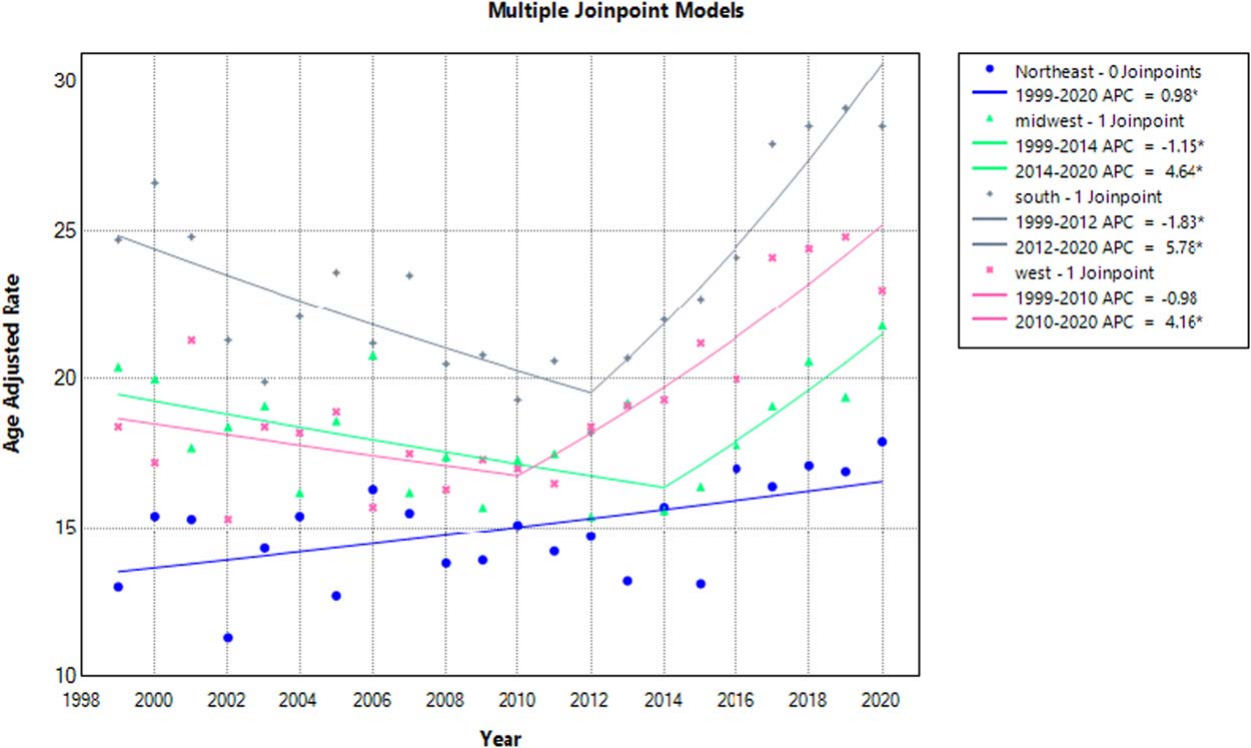
Mortality rates for malignant neoplasms of bone and articular cartilage, stratified by census region in adults aged 65 years or older in the United States between 1999 and 2020, along with the associated age-adjusted mortality rates (AAMRs) per 1 000 000. The * denotes the annual percentage change (APC) that was found to be statistically significant at α=0.05.

The mortality rates for each census region were ranked as follows: The South region had the highest AAMR (23.5; 95% CI: 23.0–24.0), followed by the West (AAMR; 19.8; 95% CI: 19.5–20.1), the Midwest (AAMR; 18.2; 95% CI: 17.6–18.8), and the Northeast (AAMR; 15.0; 95% CI: 14.5–15.6). There was a decrease in mortality trends from 1999 to 2014 (APC: −1.15; 95% CI: −3.85 to −0.21) in the Midwest, followed by an increase from 2014 to 2020 (APC; 4.64; 95% CI: 1.32–14.37). Similar trends were observed in the South region from 1999 to 2012 (APC: −1.83; 95% CI: −3.18 to −0.77), followed by an increase from 2012 to 2020 (APC; 5.78; 95% CI: 4.04–8.75). Death rates also decreased from 1999 to 2010 (APC: −0.98; 95% CI: −4.50 to 0.59) in the West and continued to increase until 2020 (APC; 4.16; 95% CI: 2.73–7.55) (Fig. 5, Supplementary Table 7, http://links.lww.com/MS9/A609).

**Figure 6:**
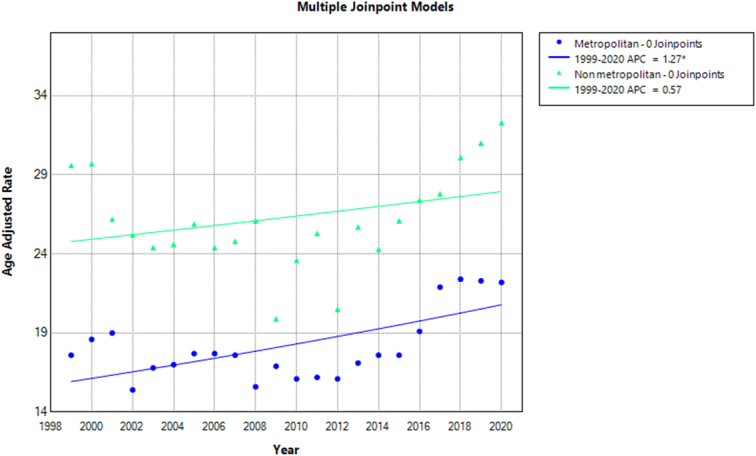
Mortality rates for malignant neoplasms of bone and articular cartilage, stratified by urbanization in adults aged 65 years or older in the United States between 1999 and 2020, along with the associated age-adjusted mortality rates (AAMRs) per 1 000 000. The * denotes the annual percentage change (APC) that was found to be statistically significant at α=0.05.

Mortality rates were also analyzed by urbanization and showed a decrease in death rates from 1999 to 2012 (APC: −0.81; 95% CI: −2.22 to 0.11) in metropolitan areas, followed by an increase from 2012 to 2020 (APC: 4.76; 95% CI: 3.24–7.61). Similar trends were observed in nonmetropolitan areas, with a decrease in death rates from 1999 to 2012 (APC: −1.65; 95% CI: −3.01 to −0.70), followed by an increase in rates until 2020 (APC: 4.66; 95% CI: 2.96–7.68). However, the total age-adjusted mortality rate (AAMR) was higher in nonmetropolitan areas (AAMR: 26.2; 95% CI: 25.5–27) compared to metropolitan areas (AAMR: 18.4; 95% CI: 18.1–18.7) during the study period (Fig. 6, Supplementary Tables 3, 8, http://links.lww.com/MS9/A609).

## Discussion

Throughout the course of two decades, the mortality data from the Centers for Disease Control and Prevention were carefully studied, and several significant discoveries were found. First, our first research indicated that between 1999 and 2020, the death rate rose. But with additional investigation, a more intricate pattern emerged. This analysis identified a unique trend. Malignant neoplasm-related mortality showed a tendency towards decline from 1999 to 2012, but then began to grow, with 2020 showing the highest AAMR. Second, compared to females, males had a greater death rate and a steeper mortality trend until 2020. Black people in New Hampshire had a higher AAMR than white people, American Indians or Alaska Natives, Hispanic or Latino people, and people from Asian or Pacific islands. We also observed significant differences between different US regions. The West, Midwest, and Northeast were the next regions with the highest AAMR, after the South. Moreover, until 2012, metropolitan areas had lower AAMR values; following that, AAMR values progressively rose in both metropolitan and nonmetropolitan areas, with metropolitan areas ultimately overtaking nonmetropolitan areas in the end. This phenomenon can be attributed to several factors, including the widespread adoption of advanced diagnostic techniques, such as CT and MRI imaging, which have enhanced detection capabilities and contributed to more accurate diagnoses. Furthermore, improvements in online database reporting facilities have likely led to higher reporting rates, providing a more comprehensive picture of the disease burden. The aging population is also a significant contributor to the rising trend, as the majority of bone tumor cases occur in older adults. While some argue that vitamin D deficiency, resulting from inadequate sun exposure, may play a role, it is also plausible that environmental factors, such as radiation and pollution, which are more prevalent in urban settings, may be contributing to the increased incidence of bone tumors in metropolitan areas^18^. States in the top 90th percentile had greater AAMR values than those in the 10th percentile, indicating that AAMR values varied among states as well. These results have important and evident implications for public health programs (Fig. 3, Supplementary Table 6, http://links.lww.com/MS9/A609).

Our findings highlight notable variances among racial and ethnic demographics, with African Americans individuals exhibiting the highest and NH-Asian individuals the lowest AAMRs. The African nations of Nigeria, Uganda, and Sudan have also reported high rates of osteosarcoma, which has been connected to a hereditary propensity based on lineage^19^. Additionally, it has been observed that individuals with African ancestry have twice as many harmful germline TP53 mutations (9% vs. 4% of cases) as cases with other ancestries^7,20^. A concerted effort to improve recruitment is necessary to better understand the etiology of osteosarcoma in instances of African ancestry, as African Americans have historically been underrepresented in genetic etiology research. Furthermore, assessing discrepancies within establishments and implementing culturally customized, interdisciplinary interventions could assist in reducing racial inequalities. For instance, Siegel *et al*.^21^ found significant disparities in cancer treatment, with Black patients being less likely to receive surgery or radiotherapy compared to White patients, which directly impacts survival outcomes.

We confirmed that females have a survival advantage over males^20,22,23^, and recent studies have identified male sex as an independent risk factor for decreased survival^24,25^. Although the exact cause of the gender disparity in survival is unknown, it may have something to do with endogenous sex hormones as well as variations in the pharmacokinetics and responsiveness to medicinal interventions^26^. This could also be partly attributed to biological differences such as hormonal influences and differences in tumor biology between sexes. Our investigation revealed that nonmetropolitan areas exhibit a higher incidence of malignant bone neoplasm-related mortality when compared to their metropolitan counterparts^27^. This disparity may be attributed to the lower socioeconomic status prevalent in rural settings and a shortage of primary care physicians and oncologists serving these regions. Over the period from 2002 to 2015, nonmetropolitan areas experienced a decline in the number of primary care physicians that was twice as significant as that observed in urban areas^28^. Similarly, these rural areas often face challenges in accessing specialized medical care, including oncological expertise, potentially contributing to the observed discrepancies in malignant bone neoplasm-related mortality rates between different geographical regions^29^.

Furthermore, we observed notable geographic variances in malignant bone neoplasm-related mortality, with the South region displaying the highest-burden compared to other US regions. These differences may be attributed to variations in the incidence rates of specific primary malignant bone tumors among diverse ethnic groups; for example, Ewing sarcoma is predominantly prevalent among Caucasian populations but less common among individuals of African descent^30^. Additionally, the significant regional gap could be influenced by variances in healthcare infrastructure, access to specialized oncology services, screening initiatives, and public health efforts. Our findings highlight the necessity of undertaking extensive population-based studies in these areas to pinpoint crucial factors responsible for the observed disparities, while also emphasizing the importance of implementing targeted public health measures, such as increasing access to cancer care in financially disadvantaged regions, improving screening programs, and raising awareness among both healthcare professionals and the general public about these less common yet life-threatening cancers.

## Limitations

Our research has certain limitations that need to be acknowledged. One limitation is that our study was retrospective. Retrospective studies typically depend on existing records, including data from specific age groups, geographic regions, and years, which may result in selection bias, incomplete, inaccurate, or inconsistent results. When data is missing, it can result in biased outcomes and negatively impact the study’s validity when compared to prospective studies. Additionally, the CDC Database, while a valuable resource, lacks specific clinical data related to treatment plans, histologic response to chemotherapy, and molecular pathological features. Furthermore, the data was not noteworthy for Alaska native and Pacific Islanders, limiting race stratification results in our study. However, our extensive, population-based investigation has defined significant incidence and survival patterns. Despite the heterogeneity of malignant neoplasm of bone and articular cartilage, we were able to distinguish variations between groups, especially among racial and ethnic minorities, which are often understudied due to small sample sizes. Improved knowledge of etiology for all age groups and racial/ethnic groups could lead to better risk assessment and tailored care, resulting in improved patient outcomes compared to the past 30 years.

## Conclusion

This research examines mortality trends of malignant neoplasms of bone and articular cartilage in individuals aged 65 and older from 1999 to 2020. The analysis reveals an initial decline in mortality rates, followed by an increase, peaking in 2020. Various factors, including sex, race/ethnicity, geographic location, and urbanization, significantly influence these trends. Males, NH Black or African Americans, southern state residents, and individuals in nonmetropolitan areas exhibit higher mortality rates, highlighting disparities needing public health intervention. Furthermore, additional research is necessary to emphasize this issue in the near future. The study underscores the need for targeted public health strategies, such as improving access to cancer care in disadvantaged areas, enhancing screening initiatives, and raising awareness of these rare but fatal cancers among healthcare professionals and the public. Identifying variations and their causes is crucial for developing strategies to reduce mortality and improve outcomes for affected populations.

## Ethical approval

This study was exempted from the institutional review board’s approval because it uses publicly available data that is deidentified.

## Consent

No consent was required for this review.

## Source of funding

The authors received no extramural funding for the study.

## Author contribution

A.H.: conceptualization, data curation, formal analysis, and writing – original draft; R.M.U.N. and M.A.S.: writing – original draft; H.A.U.R. and S.M.S.A.: data curation and writing – original draft; D.A. and L.K.: writing – original draft; M.A.S.: conceptualization and writing – original draft; M.S.M., S.R., and A.R.: writing – original draft; K.A.H.M.A.: writing, editing – original draft, and visualization.

## Conflicts of interest disclosure

The authors declare no conflicts of interest.

## Research registration unique identifying number (UIN)

This is not needed as this paper is a comprehensive review and not a systematic review or meta-analysis.

## Guarantor

Khabab Abbasher Hussien Mohamed Ahmed.

## Data availability statement

The dataset supporting the conclusions of this article is included in this article.

## Provenance and peer review

Externally peer-reviewed, not commissioned.

## Supplementary Material
