# Supplementary material for: Trends in malignant neoplasm of bone and articular cartilage related mortality among older adults in United States (1999–2020)
Source: Ann Med Surg (Lond). 2024 Sep 30;86(11):6645–52. doi: 10.1097/MS9.0000000000002629 (PMC11543209; doi:10.1097/MS9.0000000000002629)
Supplement: Supplementary file 1 [file ms9-86-6645-s001.docx]

**Supplemental Table 1** Malignant neoplasm of bone and articular cartilage related Deaths, Stratified by Sex and Race in the United States, 1999 to 2020.

| **Deaths** | | | | | | | | | |
| --- | --- | --- | --- | --- | --- | --- | --- | --- | --- |
| **Year** | **Overall** | **Women** | **Men** | **NH White** | **NH Black or African American** | **NH Asian or Pacific Islander** | **NH American Indian or Alaska Native** | **Hispanic or Latino** | **Population** |
| 1999 | 691 | 351 | 340 | 605 | 46 | Unknown | Unknown | 31 | 34797841 |
| 2000 | 725 | 376 | 349 | 629 | 52 | Unknown | Unknown | 29 | 34991753 |
| 2001 | 719 | 366 | 353 | 616 | 59 | 13 | Unknown | 26 | 35290291 |
| 2002 | 618 | 322 | 296 | 524 | 57 | Unknown | Unknown | 27 | 35522207 |
| 2003 | 659 | 333 | 326 | 568 | 54 | Unknown | Unknown | 23 | 35863529 |
| 2004 | 672 | 363 | 309 | 574 | 56 | Unknown | Unknown | 35 | 36203319 |
| 2005 | 711 | 340 | 371 | 605 | 54 | Unknown | Unknown | 41 | 36649798 |
| 2006 | 712 | 369 | 343 | 605 | 62 | Unknown | Unknown | 32 | 37164107 |
| 2007 | 723 | 341 | 382 | 606 | 64 | 12 | Unknown | 37 | 37825711 |
| 2008 | 686 | 358 | 328 | 584 | 51 | 12 | Unknown | 36 | 38777621 |
| 2009 | 695 | 331 | 364 | 576 | 62 | 14 | Unknown | 41 | 39623175 |
| 2010 | 707 | 315 | 392 | 593 | 56 | 12 | Unknown | 37 | 40267984 |
| 2011 | 740 | 340 | 400 | 617 | 70 | 10 | Unknown | 40 | 41394141 |
| 2012 | 730 | 326 | 404 | 603 | 64 | 13 | Unknown | 45 | 43145356 |
| 2013 | 820 | 392 | 428 | 678 | 65 | 12 | Unknown | 62 | 44704074 |
| 2014 | 850 | 385 | 465 | 712 | 61 | 20 | Unknown | 48 | 46243211 |
| 2015 | 894 | 405 | 489 | 731 | 68 | 18 | Unknown | 70 | 47760852 |
| 2016 | 987 | 445 | 542 | 780 | 104 | 26 | Unknown | 71 | 49244195 |
| 2017 | 1136 | 488 | 648 | 927 | 106 | 34 | Unknown | 64 | 50858679 |
| 2018 | 1209 | 528 | 681 | 973 | 124 | 27 | Unknown | 75 | 52431193 |
| 2019 | 1238 | 532 | 706 | 984 | 115 | 39 | Unknown | 90 | 54058263 |
| 2020 | 1283 | 546 | 737 | 1002 | 138 | 33 | Unknown | 104 | 55659365 |
| **Total** | 18205 | 8552 | 9653 | 15092 | **1,588** | 343 | 83 | 1064 | 928476665 |

**Supplemental Table 2** Malignant neoplasm of bone and articular cartilage related Mortality, Stratified by Place of Death in the United States, 1999 to 2020.

| **Deaths** | | | | | |  |
| --- | --- | --- | --- | --- | --- | --- |
| **Year** | **Medical Facility** | **Nursing Home/Long-term Care Facility** | **Hospices** | **Home** | **Other** |  |
|  |  |  |  |  |  |  |
| 1999 | 149 | 166 | Missing | 270 | 29 |  |
| 2000 | 169 | 163 | Missing | 239 | 47 |  |
| 2001 | 187 | 170 | Missing | 248 | 35 |  |
| 2002 | 122 | 156 | Missing | 225 | 47 |  |
| 2003 | 151 | 148 | Missing | 231 | 42 |  |
| 2004 | 126 | 141 | Missing | 283 | 45 |  |
| 2005 | 144 | 158 | 24 | 280 | 33 |  |
| 2006 | 126 | 159 | 29 | 282 | 36 |  |
| 2007 | 158 | 171 | 30 | 264 | 25 |  |
| 2008 | 116 | 140 | 40 | 268 | 36 |  |
| 2009 | 114 | 165 | 40 | 253 | 41 |  |
| 2010 | 108 | 172 | 55 | 265 | 35 |  |
| 2011 | 119 | 164 | 57 | 290 | 47 |  |
| 2012 | 138 | 145 | 53 | 273 | 49 |  |
| 2013 | 116 | 161 | 61 | 355 | 50 |  |
| 2014 | 104 | 171 | 109 | 354 | 36 |  |
| 2015 | 119 | 155 | 114 | 389 | 37 |  |
| 2016 | 130 | 184 | 137 | 409 | 40 |  |
| 2017 | 171 | 231 | 123 | 465 | 53 |  |
| 2018 | 141 | 213 | 139 | 543 | 56 |  |
| 2019 | 154 | 214 | 138 | 559 | 63 |  |
| 2020 | 142 | 207 | 112 | 623 | 78 |  |
| **Total** | 3004 | 3754 | 1265 | 7368 | 960 |  |
|  |  |  |  |  |  |  |

**Supplemental Table 3** Annual percent change (APC) of Malignant neoplasm of bone and articular cartilage related Mortality, related Age-Adjusted Mortality Rates in the United States, 1999 to 2020

| **Year Interval** | **APC (95% CI)** |
| --- | --- |
| **Overall** | |
| 1999-2012 | -1.12* ( -2.44 to -0.22) |
| 2012-2020 | 4.73*(2.99 to 8.49) |
| **Male** | |
| 1999-2012 | -0.70 (-3.51 to 0.46) |
| **2012-2020** | 5.05*(2.61 to 13.30) |
| **Female** | |
| 1999-2012 | -1.78* (-3.50 to -0.82) |
| **2012-2020** | 4.09* (2.04 to 9.43) |
| **NH White** | |
| 1999-2012 | -1.02*( -2.15 to -0.22) |
| **2012-2020** | 4.70* (3.24 to 7.15) |
| **NH Black or African American** | |
| 1999-2014 | -0.48 (-9.79 to 1.57) |
| **2014-2020** | 7.80* (2.09 to 25.05) |
| **NH American Indian or Alaska Native** | |
| 1999-2012 | Unknown |
| **2012-2020** | Unknown |
| **Hispanic or Latino** | |
| 1999-2012 | -2.13 (-15.48 to 0.91) |
| **2012-2020** | 3.72*(1.36 to 14.60) |
| **NH Asian or Pacific Islander** | |
| **2014-2020** | 2.28 (-7.99 to 16.58) |
| **Nonmetropolitan areas** | |
| 1999-2012 | -1.65* (-3.01 to -0.70) |
| **2012-2020** | 4.66* (2.96 to 7.68) |
| **Metropolitan area** | |
| 1999-2012 | -0.81 (-2.22 to 0.11) |
| **2012-2020** | 4.76* (3.24 to 7.61) |
| **Northeast region** | |
| 1999-2020 | 0.98*(0.19 to 1.87) |
| **Midwest region** | |
| 1999-2014 | -1.15* (-3.85 to -0.21) |
| **2014-2020** | 4.64* (1.32 to 14.37) |
| **South region** | |
| 1999-2012 | -1.83*( -3.18 to -0.77) |
| **2012-2020** | 5.78* (4.04 to 8.75) |
| **West region** | |
| 1999-2010 | -0.98 (-4.50 to 0.59) |
| **2010-2020** | 4.16* (2.73 to 7.55) |
| APC = annual percent change; NH = non-Hispanic; * Indicates that the annual percentage change (APC) is significantly different from zero at α = 0.05. AAMR = age-adjusted mortality rate. |  |
|  |  |

**Supplemental Table 4** Overall and Sex‐Stratified Malignant neoplasm of bone and articular cartilage –related Age-Adjusted Mortality Rates in the United States, 1999 to 2020.

| **Age-Adjusted Rate (95% CI)** | | | |
| --- | --- | --- | --- |
| **Year** | **Male** | **Female** | **Overall** |
| 1999 | 25.6 (22.9-28.4) | 16.6 (14.9-18.4) | 20 (18.5-21.5) |
| 2000 | 26.1 (23.3-28.9) | 17.5 (15.7-19.3) | 20.8 (19.2-22.3) |
| 2001 | 25.4 (22.7-28.1) | 16.8 (15.1-18.5) | 20.4 (18.9-21.9) |
| 2002 | 21.6 (19.1-24.1) | 14.8 (13.2-16.5) | 17.4 (16.0-18.7) |
| 2003 | 23.0 (20.5-25.6) | 15.1 (13.5-16.7) | 18.3 (16.9-19.7) |
| 2004 | 21.4 (19.0-23.8) | 16.5 (14.8-18.2) | 18.4 (17.1-19.8) |
| 2005 | 25.3 (22.7-27.8) | 15.2 (13.6-16.9) | 19.2 (17.8-20.6) |
| 2006 | 22.8 (20.4-25.2) | 16.2 (14.6-17.9) | 19.0 (17.6-20.3) |
| 2007 | 24.9 (22.4-27.4) | 14.8 (13.2-16.4) | 18.9 (17.5-20.3) |
| 2008 | 20.8 (18.5-23.0) | 15.2 (13.6-16.8) | 17.5 (16.2-18.8) |
| 2009 | 22.6 (20.3-24.9) | 14.0 (12.4-15.5) | 17.4 (16.1-18.7) |
| 2010 | 24.0 (21.6-26.4) | 13.1 (11.7-14.6) | 17.5 (16.2-18.8) |
| 2011 | 23.5 (21.2-25.9) | 13.7 (12.2-15.1) | 17.8 (16.5-19.1) |
| 2012 | 22.8 (20.6-25.1) | 12.6 (11.2-14.0) | 16.9 (15.7-18.2) |
| 2013 | 23.3 (21.0-25.5) | 15.1 (13.6-16.6) | 18.6 (17.3-19.9) |
| 2014 | 24.4 (22.2-26.7) | 14.6 (13.2-16.1) | 18.8 (17.5-20.0) |
| 2015 | 25.0 (22.8-27.3) | 14.8 (13.3-16.3) | 19.1 (17.8-20.4) |
| 2016 | 26.8 (24.5-29.1) | 15.9 (14.4-17.4) | 20.5 (19.2-21.8) |
| 2017 | 31.1 (28.6-33.5) | 17.0 (15.4-18.5) | 22.9 (21.6-24.3) |
| 2018 | 31.5 (29.1-33.9) | 17.9 (16.4-19.5) | 23.8 (22.4-25.1) |
| 2019 | 31.9 (29.5-34.3) | 17.8 (16.3-19.3) | 23.8 (22.4-25.1) |
| 2020 | 31.7 (29.4-34.0) | 17.8 (16.3-19.3) | 23.8 (22.5-25.2) |
| Total | 25.7 (25.2-26.2) | 15.6 (15.3-15.9) | 19.8 (19.5-20.1) |

**Supplemental Table 5** Malignant neoplasm of bone and articular cartilage –related Age-Adjusted Mortality Rates , Stratified by Race in the United States, 1999 to 2020.

| **Year** | **NH WHITE** | **NH Black or African American** | **NH American Indian or Alaska Native** | **Hispanic or Latino** | **NH Asian or Pacific Islander** |
| --- | --- | --- | --- | --- | --- |
| 1999 | 20.5 (18.90-22.2) | 16.8 (12.30-22.40) | Unknown | 21.6 (14.6-30.9) | Unknown |
| 2000 | 21.2 (19.50-22.8) | 19.1 (14.30-25.00) | Unknown | 18.1 (12-26.2) | Unknown |
| 2001 | 20.6 (19.00-22.2) | 20.9 (15.90-27.00) | Unknown | 15.9 (10.3-23.5) | Unknown |
| 2002 | 17.4 (15.90-18.9) | 20.2 (15.20-26.20) | Unknown | 15.6 (10.2-22.8) | Unknown |
| 2003 | 18.7 (17.20-20.3) | 19.1 (14.30-25.00) | Unknown | 13.1 (8.2-19.8) | Unknown |
| 2004 | 18.9 (17.30-20.4) | 19.5 (14.70-25.40) | Unknown | 17.7 (12.3-24.7) | Unknown |
| 2005 | 19.6 (18.10-21.2) | 18.3 (13.70-24.00) | Unknown | 19.7 (14.1-26.9) | Unknown |
| 2006 | 19.5 (17.90-21) | 20.6 (15.80-26.50) | Unknown | 14.6 (9.9-20.7) | Unknown |
| 2007 | 19.2 (17.70-20.7) | 20.9 (16.10-26.80) | Unknown | 16.2 (11.3-22.4) | Unknown |
| 2008 | 18.2 (16.70-19.6) | 16.1 (11.90-21.20) | Unknown | 15.6 (10.9-21.7) | Unknown |
| 2009 | 17.6 (16.10-19) | 19.1 (14.60-24.50) | Unknown | 16.0 (11.4-21.8) | Unknown |
| 2010 | 18.0 (16.50-19.4) | 16.6 (12.50-21.60) | Unknown | 13.5 (9.5-18.7) | Unknown |
| 2011 | 18.3 (16.80-19.8) | 20.6 (16.00-26.00) | Unknown | 14.0 (9.9-19.1) | Unknown |
| 2012 | 17.3 (16.00-18.7) | 17.8 (13.70-22.80) | Unknown | 14.9 (10.8-20.0) | Unknown |
| 2013 | 19.3 (17.80-20.7) | 17.3 (13.30-22.10) | Unknown | 20.2 (15.4-25.9) | Unknown |
| 2014 | 19.8 (18.30-21.2) | 16.0 (12.20-20.60) | Unknown | 14.9 (11-19.8) | 15 (7.00-17.70) |
| 2015 | 19.7 (18.30-21.2) | 17.3 (13.30-22.00) | Unknown | 19.7 (15.3-24.9) | Unknown |
| 2016 | 20.6 (19.20-22.1) | 24.9 (20.00-29.70) | Unknown | 18.8 (14.6-23.8) | 11.5 (8.10-18.40) |
| 2017 | 24.0 (22.40-25.5) | 24.4 (19.70-29.10) | Unknown | 16.0 (12.3-20.5) | 14.8 (10.20-20.80) |
| 2018 | 24.6 (23.00-26.1) | 27.3 (22.40-32.20) | Unknown | 18.1 (14.2-22.7) | 11.6 (7.60-17.10) |
| 2019 | 24.5 (22.90-26) | 23.1 (18.80-27.40) | Unknown | 21.0 (16.8-25.8) | 15.9 (11.30-21.90) |
| 2020 | 24.3 (22.80-25.8) | 28.0 (23.30-32.80) | Unknown | 22.3 (18-26.7) | 12.4 (8.40-17.40) |
| **Total** | 20.2 (19.90-20.5) | 20.6 (19.6-21.6) | 19.1 (15.1-23.8) | 17.5 (16.5-18.6) | 10.5 (9.30-11.6) |

**Supplemental Table 6** Malignant neoplasm of bone and articular cartilage –related Age-Adjusted Mortality, Stratified by States in the United States, 1999 to 2020.

| **State** | **Age-Adjusted Rate (95% CI)** |
| --- | --- |
| Alabama | 33.8 (30.8-36.8) |
| Alaska | Unreliable (5.7-18.4) |
| Arizona | 19.7 (17.8-21.7) |
| Arkansas | 49.2 (44.6-53.7) |
| California | 20.7 (19.8-21.6) |
| Colorado | 17.0 (14.7-19.3) |
| Connecticut | 13.3 (11.2-15.4) |
| Delaware | 16.2 (11.9-21.6) |
| District of Columbia | 12.1 (7.4-18.6) |
| Florida | 17.2 (16.3-18.1) |
| Georgia | 22.9 (20.9-24.9) |
| Hawaii | 12.0 (9.0-15.7) |
| Idaho | 17.1 (13.4-21.5) |
| Illinois | 17.9 (16.5-19.3) |
| Indiana | 18.8 (16.8-20.7) |
| Iowa | 16.7 (14.2-19.2) |
| Kansas | 19.5 (16.6-22.4) |
| Kentucky | 27.4 (24.5-30.3) |
| Louisiana | 34.2 (31.0-37.5) |
| Maine | 15.4 (12.1-19.4) |
| Maryland | 16.2 (14.2-18.1) |
| Massachusetts | 15.4 (13.8-17.1) |
| Michigan | 20.0 (18.4-21.6) |
| Minnesota | 15.8 (13.8-17.7) |
| Mississippi | 65.0 (59.5-70.4) |
| Missouri | 21.1 (19.1-23.2) |
| Montana | 16.3 (12.2-21.2) |
| Nebraska | 20.8 (17.1-24.5) |
| Nevada | 20.7 (17.2-24.1) |
| New Hampshire | 13.4 (10.1-17.5) |
| New Jersey | 15.5 (14.0-17.0) |
| New Mexico | 24.7 (20.7-28.7) |
| New York | 14.4 (13.4-15.3) |
| North Carolina | 16.2 (14.7-17.7) |
| North Dakota | 12.8 (8.6-18.4) |
| Ohio | 16.4 (15.1-17.7) |
| Oklahoma | 26.9 (23.8-29.9) |
| Oregon | 17.0 (14.7-19.3) |
| Pennsylvania | 15.7 (14.6-16.9) |
| Rhode Island | 15.0 (11.2-19.6) |
| South Carolina | 22.2 (19.7-24.7) |
| South Dakota | 21.8 (16.6-28.0) |
| Tennessee | 22.9 (20.7-25.1) |
| Texas | 25.7 (24.4-27.0) |
| Utah | 19.9 (16.2-23.6) |
| Vermont | 16.1 (11.2-22.5) |
| Virginia | 14.7 (13.1-16.3) |
| Washington | 18.9 (16.9-20.9) |
| West Virginia | 20.3 (16.8-23.7) |
| Wisconsin | 17.8 (15.8-19.7) |
| Wyoming | 20.7 (14.2-29.0) |

**Supplemental Table 7** Malignant neoplasm of bone and articular cartilage –related Age-Adjusted Mortality Stratified by Census Region in the United States, 1999 to 2020.

| **Census Region** | **Year** | **Age-Adjusted Rate (95% CI)** |
| --- | --- | --- |
| Northeast | 1999 | 13.0 (10.5-15.9) |
| Northeast | 2000 | 15.4 (12.6-18.2) |
| Northeast | 2001 | 15.3 (12.5-18.1) |
| Northeast | 2002 | 11.3 (9.0-14.0) |
| Northeast | 2003 | 14.3 (11.6-16.9) |
| Northeast | 2004 | 15.4 (12.6-18.2) |
| Northeast | 2005 | 12.7 (10.3-15.5) |
| Northeast | 2006 | 16.3 (13.5-19.2) |
| Northeast | 2007 | 15.5 (12.7-18.3) |
| Northeast | 2008 | 13.8 (11.2-16.4) |
| Northeast | 2009 | 13.9 (11.3-16.5) |
| Northeast | 2010 | 15.1 (12.4-17.8) |
| Northeast | 2011 | 14.2 (11.6-16.8) |
| Northeast | 2012 | 14.7 (12.1-17.3) |
| Northeast | 2013 | 13.2 (10.8-15.7) |
| Northeast | 2014 | 15.7 (13.0-18.4) |
| Northeast | 2015 | 13.1 (10.6-15.5) |
| Northeast | 2016 | 17.0 (14.3-19.7) |
| Northeast | 2017 | 16.4 (13.7-19.0) |
| Northeast | 2018 | 17.1 (14.4-19.7) |
| Northeast | 2019 | 16.9 (14.3-19.6) |
| Northeast | 2020 | 17.9 (15.3-20.6) |
| Northeast | **Total** | 15.0 (14.5-15.6) |
| Midwest | 1999 | 20.4 (17.4-23.5) |
| Midwest | 2000 | 20.0 (17.0-23.1) |
| Midwest | 2001 | 17.7 (14.9-20.6) |
| Midwest | 2002 | 18.4 (15.5-21.3) |
| Midwest | 2003 | 19.1 (16.2-22.0) |
| Midwest | 2004 | 16.2 (13.5-18.9) |
| Midwest | 2005 | 18.6 (15.7-21.5) |
| Midwest | 2006 | 20.8 (17.7-23.8) |
| Midwest | 2007 | 16.2 (13.6-18.9) |
| Midwest | 2008 | 17.4 (14.7-20.1) |
| Midwest | 2009 | 15.7 (13.1-18.3) |
| Midwest | 2010 | 17.3 (14.6-20.0) |
| Midwest | 2011 | 17.5 (14.8-20.2) |
| Midwest | 2012 | 15.4 (12.9-17.9) |
| Midwest | 2013 | 19.2 (16.5-22.0) |
| Midwest | 2014 | 15.6 (13.1-18.0) |
| Midwest | 2015 | 16.4 (13.9-19.0) |
| Midwest | 2016 | 17.8 (15.2-20.4) |
| Midwest | 2017 | 19.1 (16.5-21.7) |
| Midwest | 2018 | 20.6 (17.9-23.3) |
| Midwest | 2019 | 19.4 (16.8-22.0) |
| Midwest | 2020 | 21.8 (19.0-24.5) |
| Midwest | **Total** | 18.2 (17.6-18.8) |
| South | 1999 | 24.7 (21.9-27.5) |
| South | 2000 | 26.6 (23.7-29.5) |
| South | 2001 | 24.8 (22.0-27.6) |
| South | 2002 | 21.3 (18.7-23.8) |
| South | 2003 | 19.9 (17.4-22.3) |
| South | 2004 | 22.1 (19.5-24.7) |
| South | 2005 | 23.6 (21.0-26.3) |
| South | 2006 | 21.2 (18.8-23.7) |
| South | 2007 | 23.5 (20.9-26.1) |
| South | 2008 | 20.5 (18.1-22.8) |
| South | 2009 | 20.8 (18.5-23.2) |
| South | 2010 | 19.3 (17.0-21.6) |
| South | 2011 | 20.6 (18.3-22.9) |
| South | 2012 | 18.2 (16.1-20.3) |
| South | 2013 | 20.7 (18.5-23.0) |
| South | 2014 | 22.0 (19.8-24.3) |
| South | 2015 | 22.7 (20.4-25.0) |
| South | 2016 | 24.1 (21.8-26.4) |
| South | 2017 | 27.9 (25.4-30.3) |
| South | 2018 | 28.5 (26.1-30.9) |
| South | 2019 | 29.1 (26.7-31.5) |
| South | 2020 | 28.5 (26.1-30.8) |
| South | **Total** | 23.5 (23.0-24.0) |
| West | 1999 | 18.4 (15.2-21.7) |
| West | 2000 | 17.2 (14.1-20.3) |
| West | 2001 | 21.3 (17.9-24.7) |
| West | 2002 | 15.3 (12.4-18.1) |
| West | 2003 | 18.4 (15.3-21.6) |
| West | 2004 | 18.2 (15.1-21.2) |
| West | 2005 | 18.9 (15.8-22.0) |
| West | 2006 | 15.7 (12.9-18.5) |
| West | 2007 | 17.5 (14.6-20.4) |
| West | 2008 | 16.3 (13.5-19.1) |
| West | 2009 | 17.3 (14.4-20.1) |
| West | 2010 | 17.0 (14.2-19.8) |
| West | 2011 | 16.5 (13.9-19.2) |
| West | 2012 | 18.4 (15.6-21.1) |
| West | 2013 | 19.1 (16.3-21.9) |
| West | 2014 | 19.3 (16.5-22.1) |
| West | 2015 | 21.2 (18.4-24.0) |
| West | 2016 | 20.0 (17.3-22.7) |
| West | 2017 | 24.1 (21.2-27.1) |
| West | 2018 | 24.4 (21.5-27.3) |
| West | 2019 | 24.8 (21.9-27.7) |
| West | 2020 | 23.0 (20.3-25.7) |
| West | **Total** | 19.6 (19.0-20.2) |
| **Total** | **Total** | 19.8 (19.5-20.1) |

**Supplemental Table 8** Malignant neoplasm of bone and articular cartilage -related Age-Adjusted Mortality Rates , Stratified by Urban-Rural Classification in the United States, 1999 to 2020

| **Age-Adjusted Rate (95% CI)** | | |
| --- | --- | --- |
| **Year** | **metropolitan** | **nonmetropolitan** |
| 1999 | 17.6 (16.1-19.2) | 29.6 (25.5-33.8) |
| 2000 | 18.6 (17.1-20.2) | 29.7 (25.5-33.8) |
| 2001 | 19.0 (17.4-20.6) | 26.2 (22.3-30) |
| 2002 | 15.4 (14-16.9) | 25.2 (21.5-29) |
| 2003 | 16.8 (15.4-18.3) | 24.4 (20.7-28.1) |
| 2004 | 17.0 (15.5-18.5) | 24.6 (20.9-28.3) |
| 2005 | 17.7 (16.2-19.2) | 25.9 (22.1-29.7) |
| 2006 | 17.7 (16.2-19.2) | 24.4 (20.7-28) |
| 2007 | 17.6 (16.1-19.1) | 24.8 (21.2-28.5) |
| 2008 | 15.6 (14.2-17) | 26.1 (22.4-29.8) |
| 2009 | 16.9 (15.5-18.3) | 19.9 (16.7-23.2) |
| 2010 | 16.1 (14.8-17.5) | 23.6 (20.1-27.2) |
| 2011 | 16.2 (14.8-17.6) | 25.3 (21.7-28.9) |
| 2012 | 16.1 (14.8-17.5) | 20.5 (17.3-23.7) |
| 2013 | 17.1 (15.7-18.4) | 25.7 (22.1-29.3) |
| 2014 | 17.6 (16.2-18.9) | 24.3 (20.9-27.8) |
| 2015 | 17.6 (16.3-19) | 26.1 (22.6-29.7) |
| 2016 | 19.1 (17.7-20.4) | 27.4 (23.8-31) |
| 2017 | 21.9 (20.5-23.3) | 27.8 (24.2-31.4) |
| 2018 | 22.4 (21-23.8) | 30.1 (26.5-33.8) |
| 2019 | 22.3 (20.8-23.7) | 31.0 (27.3-34.7) |
| 2020 | 22.2 (20.8-23.6) | 32.3 (28.6-36.1) |
| total | 18.4(18.1-18.7) | 26.2(25.5-27) |
